# Endobronchial chondroma with ossification mimicking chronic obstructive pulmonary disease: a case report

**DOI:** 10.3389/fmed.2025.1623910

**Published:** 2025-09-12

**Authors:** Xiaoling Huang, Miaomiao Xiong, Zhiyong Jia, Qijuan Ran, Ping Tang, Shicai Zhao, Min Xiao

**Affiliations:** ^1^Department of Respiratory Medicine, Sichuan Provincial People’s Hospital East Sichuan Hospital & Dazhou First People’s Hospital, Dazhou, China; ^2^Department of Laboratory Medicine, Xuanhan People’s Hospital, Dazhou, China; ^3^Department of Respiratory Medicine, Dazhou Central Hospital, Dazhou, China; ^4^Department of Laboratory Medicine, Sichuan Provincial People’s Hospital East Sichuan Hospital & Dazhou First People’s Hospital, Dazhou, China

**Keywords:** chronic obstructive pulmonary disease, endobronchial chondroma, Department of Respiratory Medicine, the elderly, case report

## Abstract

**Background:**

Endobronchial chondroma is a rare benign mesenchymal tumour, and its etiology remains not entirely understood. Due to the tumour’s slow growth rate, the early stages of the disease often present with atypical or nonspecific clinical symptoms. Consequently, most cases are discovered incidentally during examinations. However, when the tumour enlarges to partially or completely obstruct the bronchus or exerts pressure on adjacent structures, patients may present with a range of respiratory symptoms including fever, cough, sputum production, chest pain, stridor, dyspnea, and hemoptysis. These clinical manifestations lack specificity and can easily lead to misdiagnosis as chronic obstructive pulmonary disease (COPD), asthma, pneumonia, lung cancer and other common conditions. In some cases, they may even result in missed diagnoses. Therefore, in conjunction with relevant literature, we have summarized the diagnostic and therapeutic experiences of a case involving chronic obstructive pulmonary disease complicated by endobronchial chondroma with ossification at our hospital. We hope this will provide valuable insights for clinical practice.

**Case presentation:**

A 67-year-old male farmer with a smoking history exceeding 30 years was admitted to the hospital due to persistent cough, expectoration, asthma for the past decade, and hemoptysis lasting more than 10 days. Upon admission, the patient received treatment with ceftazidime and bromhexine hydrochloride; however, no significant improvement was observed. Fiberoptic bronchoscopy revealed a neoplasm resembling paving stones in both the trachea and main bronchus, which was subsequently confirmed as endobronchial chondroma through pathological biopsy. Unfortunately, after receiving this diagnosis, the patient was discharged from the hospital without undergoing follow-up treatment. Although he was advised to return for review in 2–3 months and consider endoscopic intervention if necessary, he did not adhere to this schedule.

**Conclusion:**

Despite being rare and lacking specific clinical manifestations or imaging characteristics, it is crucial to remain vigilant regarding potential uncommon diseases such as endobronchial chondroma in patients presenting with long-term cough, expectoration, asthma symptoms alongside a smoking history—even when common conditions like chronic obstructive pulmonary disease are initially suspected. When conventional treatments prove ineffective, timely examination via fiberoptic bronchoscopy should be conducted to prevent missed diagnoses of rare lesions like endobronchial chondroma due to symptom overlap with more prevalent diseases.

## Introduction

Benign tumours of the trachea and bronchi are quite rare, accounting for only 2% of all lung tumours. From a histological classification perspective, the most common benign tumours of the trachea and bronchi include hamartomas and papillomas, while leiomyomas, lipomas, chondromas, and neurogenic tumours are relatively uncommon (1–3). Because benign tumours usually grow slowly and lack specific clinical symptoms, tracheal benign lesions may not be diagnosed clearly for months or even years, and are prone to be misdiagnosed as malignant tumours. Chondroma originates from mesenchymal tissue and mainly occurs in the skeletal system, especially the axial skeleton. It can also occur in the soft tissue near the tendon sheath of the muscle or the tendons of the hands and feet. In the respiratory system, chondroma can involve the larynx, trachea or main bronchi, while chondroma occurring in the bronchi is even rarer (4).

Bronchial chondroma is a rare benign tumour derived from the cartilaginous tissue of the trachea, bronchi, and bronchioles. It typically presents as an oval or round mass and may exhibit lobulation (1). Chondroma is a slowly growing tumour that typically presents with an insidious onset and lacks specific clinical manifestations in its early stages. As a result, it is often incidentally detected during routine examinations. However, as the tumour enlarges, it may cause partial or complete bronchial obstruction or compress adjacent anatomical structures, leading to complications such as obstructive pneumonia, atelectasis, and pulmonary abscess. These complications may result in nonspecific symptoms including cough, sputum production, chest pain, wheezing, hemoptysis, and dyspnea. Due to the similarity of these symptoms to those of common respiratory diseases such as chronic obstructive pulmonary disease (COPD) and asthma, chondroma can be easily misdiagnosed or overlooked in clinical practice (1, 5–7). Therefore, understanding the clinical manifestations, pathological characteristics, and treatment options of endobronchial chondroma is of significant importance in enhancing clinicians’ comprehension and management capabilities regarding this condition.

## Case report

**Figure 1:**
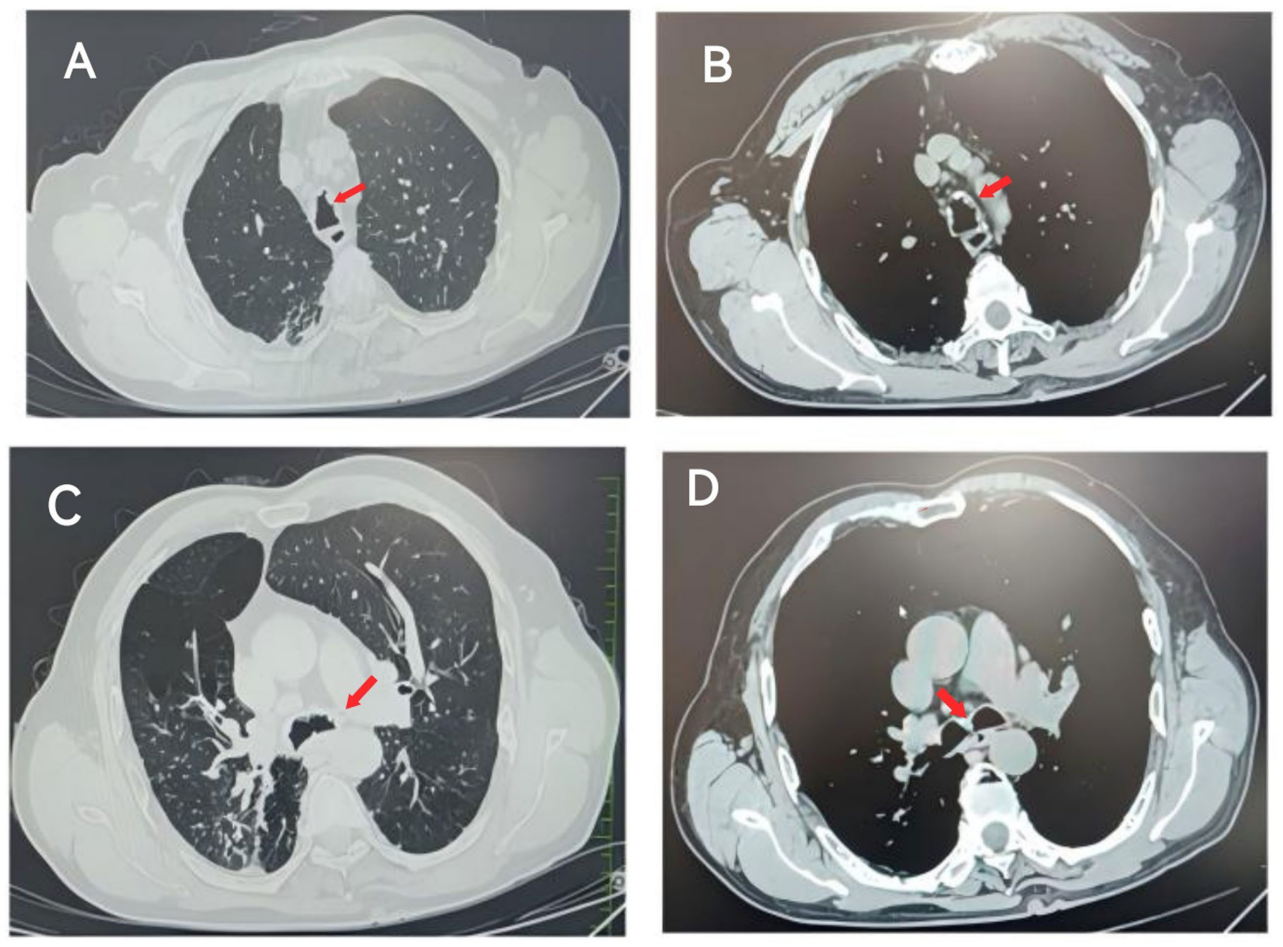
Chest CT on August 19: Panels **A,B** show scattered multiple calcifications in the tracheal wall. Panels **C,D** show scattered multiple calcifications on the left and right main bronchial walls. (Panels **A,C** are lung windows, and panels **B,D** are mediastinal windows).

**Figure 2:**
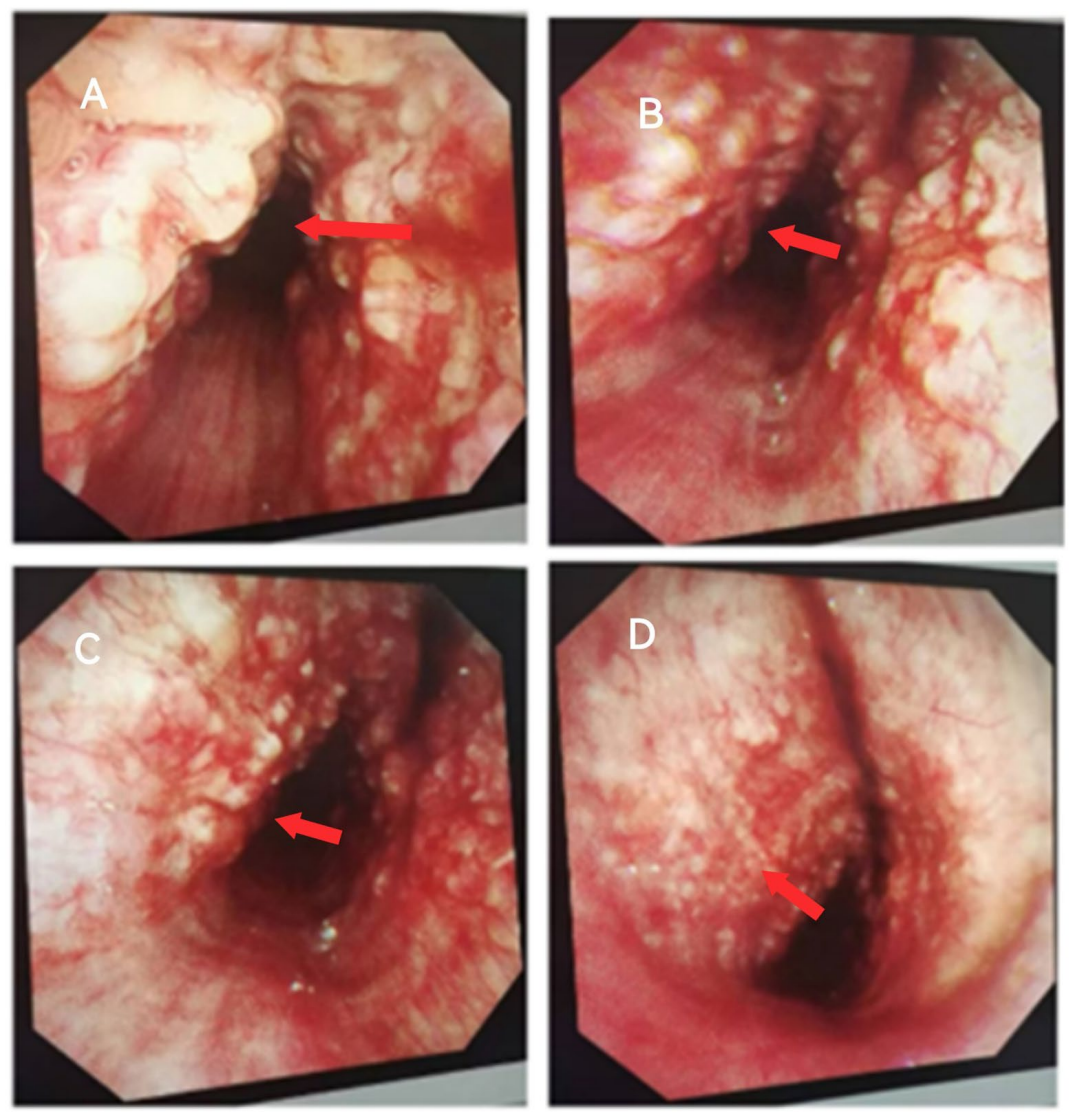
Fiberoptic bronchoscopy on August 20: Panel **A** shows a cobblestone-like neoplasm on the left and right anterior walls of the trachea with tracheal stenosis. Panels **B–D** show a paving stone-like neoplasm in the left and right main bronchus with mucosal congestion.

**Figure 3:**
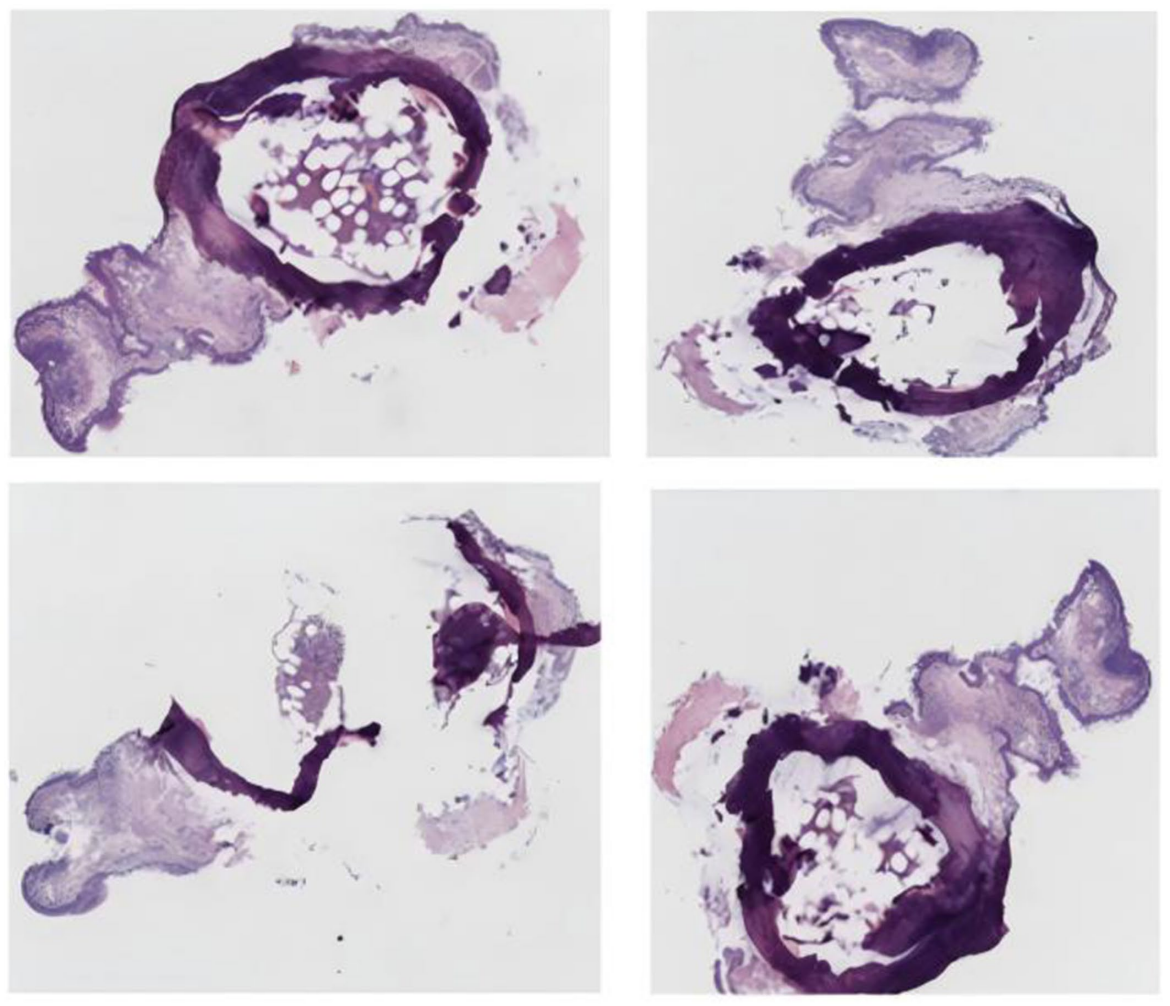
Bronchial tissue pathological biopsy image.

The patient, a 67-year-old male farmer, was admitted to the East Sichuan Hospital of Sichuan Provincial People’s Hospital and the First People’s Hospital of Dazhou on August 19, 2024 due to “cough, expectoration, asthma for 10 years, and hemoptysis for more than 10 days.” Ten years ago, the patient developed cough and sputum without obvious cause, mostly dry cough, accompanied by asthma, and then the shortness of breath increased year by year and the symptoms recurred. The symptoms could be relieved by taking drugs by himself or going to the clinic for infusion. The specific diagnosis and treatment were unknown. Personal history: “smoking history” for more than 30 years, an average of 1 pack/day. His past medical history and family history were normal. Physical examination showed barrel-shaped chest, percussion of both lungs was clear, breath sounds of both lungs were clear, and no dry or wet rales were heard. The respiratory movement of both lungs was uniform and symmetrical, without enhancement or attenuation. The tactile language tremor of both lungs was weakened, and no pleural friction was touched. Chest CT on August 19 showed diffuse small nodules in both lungs and a tree-in-bud sign in the upper lobe of the right lung, suggesting the possibility of chronic infectious disease. There was bronchiectasis in the lower lobe of the right lung, and partial atelectasis in the lower lobe of the right lung was possible. Emphysema with pulmonary bullae. There were multiple calcifications scattered in the trachea and the left and right main bronchial walls (Figure 1). Pulmonary function test on August 19 showed severe obstructive ventilatory dysfunction, FEV1%FVC <70%, significantly increased residual volume to total lung capacity, and slightly decreased diffusion function. On August 20, fiberoptic bronchoscopy revealed a cobblestone-like neoplasm in the trachea, left and right main bronchi (Figure 2). Pathology report dated 28 August: The surface of the submitted tissue was covered with pseudostratified ciliated columnar epithelium. Chondroma formation with ossification was observed in the submucosa, with bone marrow tissue in the centre, consistent with bronchial chondroma with ossification (Figure 3). Painless gastroscopy and esophagoscopy on August 29 revealed chronic atrophic gastritis. MRI plain scan of bilateral adrenal glands on August 29 showed no obvious abnormality, and scanning suggested the possibility of small cysts in the liver and bilateral renal cysts. Blood routine test showed platelet count 87 × 10^9^/L. Thyroid function: FT3 17.32 pmol/L; Blood gas analysis: PCO_2_ 48.20 mmHg PO_2_ 65.80 mmHg Lac 2.4 mmol/L PaO_2_/FiO_2_ 313; SAA, ESR, coagulation, liver and kidney function, and electrolytes were normal. No fungi, acid-fast bacilli or bacteria were found in bronchoalveolar lavage fluid.

Ceftazidime 2 g was administered intravenously every 12 h and bromhexine hydrochloride 4 mg was given twice daily after admission; however, the patient’s symptoms did not show significant improvement. Consequently, fiberoptic bronchoscopy was performed, revealing a paving stone-like neoplasm located in the trachea and bilateral main bronchi. A biopsy of the lesion was conducted, and histopathological analysis confirmed the diagnosis of endobronchial chondroma. Given the possibility of Carney syndrome, additional investigations including gastroscopy and adrenal MRI were carried out, but the findings did not support the diagnosis of Carney triad. The final diagnosis was chronic obstructive pulmonary disease complicated by endobronchial chondroma with ossification. Regrettably, despite clinical improvement and a confirmed diagnosis, the patient requested discharge and did not proceed with further treatment. At the time of discharge, the patient was advised to return for follow-up fiberoptic bronchoscopy in 2–3 months and to undergo bronchoscopic intervention if indicated. However, the patient failed to attend the scheduled outpatient follow-up.

## Discussion

Due to frequent misdiagnosis of bronchial chondroma as hamartoma, its true incidence remains uncertain (1). Compared with hamartoma, bronchial chondroma exhibits distinct pathological features, including calcified or ossified cartilage components. Histopathological examination typically reveals normal cartilage tissue with calcification, often covered by epithelium, and lacking glands, adipose tissue, smooth muscle, or other stromal elements commonly seen in hamartomas (1, 8–10). In this case, the examined tissue was covered with pseudostratified ciliated columnar epithelium, with chondroma formation accompanied by ossification observed beneath the mucosa, and bone marrow tissue present in the central region. These pathological findings were largely consistent with those previously reported in the literature.

Existing literature indicates that the clinical manifestations of endobronchial chondroma are primarily influenced by mechanical factors rather than intrinsic pathological characteristics. Chest radiography may demonstrate lobar consolidation or atelectasis, while chest CT often reveals isolated endobronchial lesions that may lead to bronchial obstruction and subsequent complications such as pneumonia, atelectasis, bronchiectasis, and fibrosis (8, 11, 12), findings that were consistent with those observed in this case. The patient, an elderly male, initially presented to the Department of Respiratory Medicine with a 10-year history of cough, expectoration, and asthma, along with more than 10 days of hemoptysis. Based on chest CT findings, pulmonary function tests, and a history of smoking, chronic obstructive pulmonary disease (COPD) and bronchiectasis were initially suspected, while rarer differential diagnoses were not considered. However, after a period of treatment, the patient continued to experience persistent wheezing and dyspnea, with suboptimal therapeutic response. A repeat chest CT scan revealed atelectasis in the right lower lobe and multiple scattered calcifications within the tracheal and bilateral main bronchial walls. Bronchoscopy identified a paving stone-like neoplasm in the trachea and main bronchi, and a biopsy was subsequently performed. Histopathological analysis confirmed the diagnosis of endobronchial chondroma with ossification.

A review of the literature indicates that isolated bronchial cartilage tumours not associated with Carney triad are extremely rare (3). Some cases of chondroma may represent one of the manifestations of Carney triad. Carney triad is a rare, non-hereditary syndrome predominantly affecting young women, characterized by the coexistence of gastric epithelioid leiomyoblastoma, extra-adrenal paraganglioma, and pulmonary chondroma (13, 14). Following the diagnosis of bronchial chondroma in this case, we considered the possibility of Carney triad and conducted gastroscopy and adrenal MRI. However, no evidence supporting this diagnosis was found. Several factors may have contributed to the delayed diagnosis in this case: (1) Although case reports of bronchial cartilage tumours exist both domestically and internationally, cases involving multiple concurrent pulmonary conditions are rarely documented. The patient presented with COPD, bronchiectasis, and atelectasis, manifesting symptoms such as cough, sputum production, wheezing, and hemoptysis—clinical features highly similar to those of common respiratory diseases, thereby complicating differential diagnosis based solely on clinical presentation; (2) Physical examination revealed only signs consistent with COPD, without other abnormal findings; (3) Clinicians lacked sufficient awareness of bronchial cartilage tumours, failing to consider this rare entity during initial evaluation and management. Although the diagnosis was delayed due to limited clinical suspicion of rare diseases, invasive diagnostic procedures were promptly initiated upon recognition of treatment failure, offering valuable insights for the management of similar clinical scenarios.

Once diagnosed, early and aggressive intervention is crucial in cases of bronchial cartilage tumours to prevent severe complications that may compromise lung architecture and significantly impair quality of life. Notably, Cao et al. (10) reported a rare complication—spontaneous pneumothorax secondary to bronchial cartilage tumours. Similarly, Grover and Zuwallack (15) described a case in which a patient with bronchial chondroma, left untreated for 4 years, eventually developed complete left lung collapse requiring extensive resection, resulting in significant pulmonary dysfunction. Salminen’s et al. (16) case report highlights the potential for recurrence and malignant transformation associated with bronchial chondromas. At the time of the first recurrence, which occurred 5 years post-initial surgery, no malignant pathological signs were detected. However, 6 years later, during the second recurrence, distinct histological features indicative of malignant chondrosarcoma were observed. A year thereafter, a third recurrence transpired, accompanied by multi-organ metastasis; tragically, the patient succumbed 14 years following the initial diagnosis. These cases underscore the critical importance of timely identification and excision of bronchial cartilage tumours by clinicians to prevent airway obstruction and related complications. While an optimal treatment regimen for bronchial cartilage tumours remains undefined, primary treatment options typically include complete resection via bronchoscopy or surgical intervention (such as video-assisted thoracoscopic surgery (VATS) or open thoracotomy). Treatment decisions should be individualized based on factors such as tumour resectability, size, type, and location to develop a tailored approach for each patient (17).

## Conclusion

Although bronchial cartilage tumours are rare and lack specific clinical manifestations and imaging features, clinicians should maintain a high index of suspicion in two key clinical scenarios: first, in patients presenting with unexplained chronic cough, sputum production, wheezing, dyspnoea, or recurrent respiratory infections, as these symptoms may be indicative of underlying bronchial tumours; second, when radiological findings reveal lobar consolidation, atelectasis, or calcifications, bronchial tumours should be considered in the differential diagnosis. However, due to the rarity of these lesions, both radiologists and clinicians often have limited awareness, which may result in missed or incorrect diagnoses and pose significant challenges to early detection and management. Therefore, when clinical presentations overlap with those of more common respiratory diseases, timely bronchoscopy serves as a crucial diagnostic tool for identifying tracheobronchial lesions, thereby reducing the likelihood of missed diagnoses. According to previous literature and the clinical experience from this case, bronchial chondromas carry potential risks of recurrence and malignant transformation. Early diagnosis and surgical resection can effectively prevent airway obstruction and associated complications. Although the patient in this case was unable to proceed with further treatment due to non-compliance, this situation underscores the critical importance of initiating timely and standardised management following diagnosis.

## Data Availability

The original contributions presented in the study are included in the article/supplementary material, further inquiries can be directed to the corresponding authors.
